# 

**Published:** 1877-10

**Authors:** 


### Books and Pamphlets Received.

Transactions of the College of Physicians of Philadalphia. Volume the Tenth. Third series, Vol. the Third. Philadelphia: Printed for the College. For Sale by Lindsay & Blakiston. 1877.

Transactions of the Kentucky State Medical Society, April, 1877. Louisville, 1877.

Practical Hints on the selection and use of the Microscope. By John Phin. Second Edition. New York: The Industrial Publication Company, 1877.

Hospitals: Their History, Organization and Construction. By W. Gill Wylie, M. D. New York: D. Appleton & Co., 1877. Buffalo: Herger & Ulbrich.

Transactions of the Association of the Alumni and Officers of the Medical Department of the University of Buffalo, for the years 1875-1876-1877.

The Physicians Visiting List for 1878. Lindsay & Blakiston.

The Popular Science Monthly for October.

Excision of the lower end of the Rectum in cases of Cancer. By John B. Roberts, M. D. Reprint from Medical and Surgical Reporter.

Pathology and Treatment of Sprains. By Richard O. Cowling, M. D.

The relations existing between Eczema and Psoriasis. By Robert Campbell, M. D.

Morphia in Childbirth. By W. T. Lusk, M. D.

Veratrum Viride. By Jno. S. Lynch, M. D.

Retarded Dilatation of Os Uteri in Labor. By Albert H. Smith, M. D., Philadelphia.

### Contents of October Number.

### ORIGINAL.

**Medical and Surgical Journal**

PUBLISHED MONTHLY,

Containing Original Articles, Reports of Medical Societies and Hospitals, Editorial, Reviews, Correspondence, Army News, etc., etc ; including the usual variety of Medical Periodical Publications. Specimen copies sent on application

Address Buffalo Medical & Surgical Journal, Buffalo, N. Y.

TERMS OF SUBSCRIPTION, $3.00 PER YEAR, IN ADVANCE.

